# Bone Marrow Involvement in Sarcoidosis: A Case Report of Granulomatous and Hematologic Complications

**DOI:** 10.1002/ccr3.72141

**Published:** 2026-03-19

**Authors:** Nina Couette, Zachary Braunstein, Daniel Jones, Jonathan E. Brammer, Arindam Singha

**Affiliations:** ^1^ Division of Rheumatology & Immunology The Ohio State University Wexner Medical Center Columbus Ohio USA; ^2^ Division of Hematology and Medical Oncology The Ohio State University Wexner Medical Center and James Comprehensive Cancer Center Columbus Ohio USA; ^3^ Department of Pathology The Ohio State University Wexner Medical Center Columbus Ohio USA; ^4^ Division of Pulmonary, Critical Care, and Sleep Medicine The Dorothy M. Davis Heart and Lung Research Institute Columbus Ohio USA

**Keywords:** aplastic anemia, bone marrow, granulomatous inflammation, hematology, sarcoidosis, T‐LGLL

## Abstract

Bone marrow involvement in sarcoidosis is rare and poorly understood. This case highlights the hematologic complexity of sarcoidosis, including its association with aplastic anemia and T‐cell large granular lymphocytic leukemia (T‐LGLL). We report a 70‐year‐old male with longstanding sarcoidosis who developed multiple hematologic complications, including bone marrow granulomatous infiltration, aplastic anemia, and T‐LGLL.

## 1 Introduction

Bone marrow involvement in sarcoidosis is a rare manifestation with an incidence of < 5% [1]. Clinical indications for obtaining a bone marrow biopsy in sarcoidosis remain poorly defined [2]. We report a case of a patient who experienced multiple hematologic complications, including aplastic anemia, T‐cell large granular lymphocytic leukemia (T‐LGLL), and granulomatous bone marrow involvement.

## 2 Case Presentation

**FIGURE 1:**
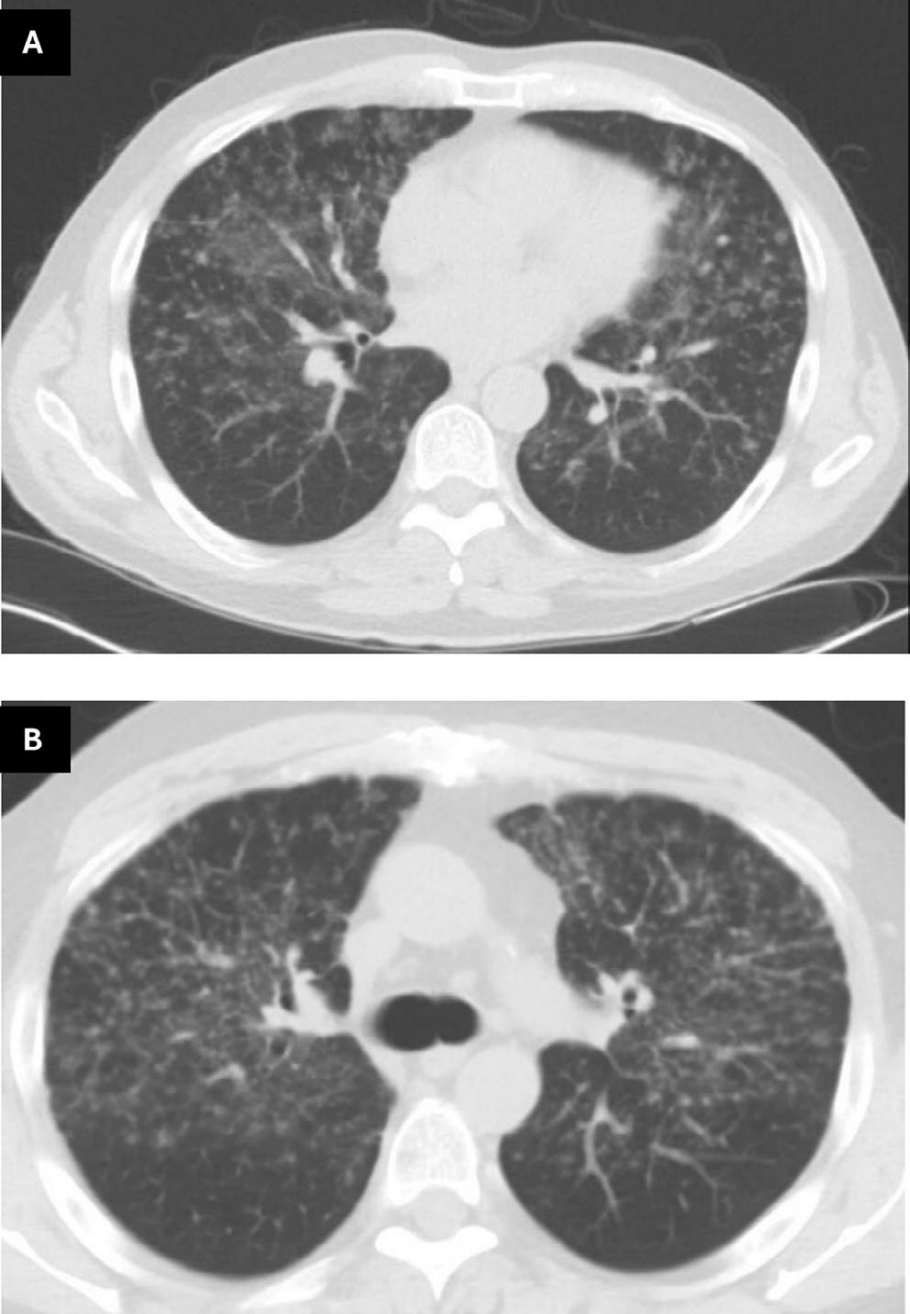
Chest CT with diffuse upper lung predominant perilymphatic micronodules.

**IMAGE 1:**
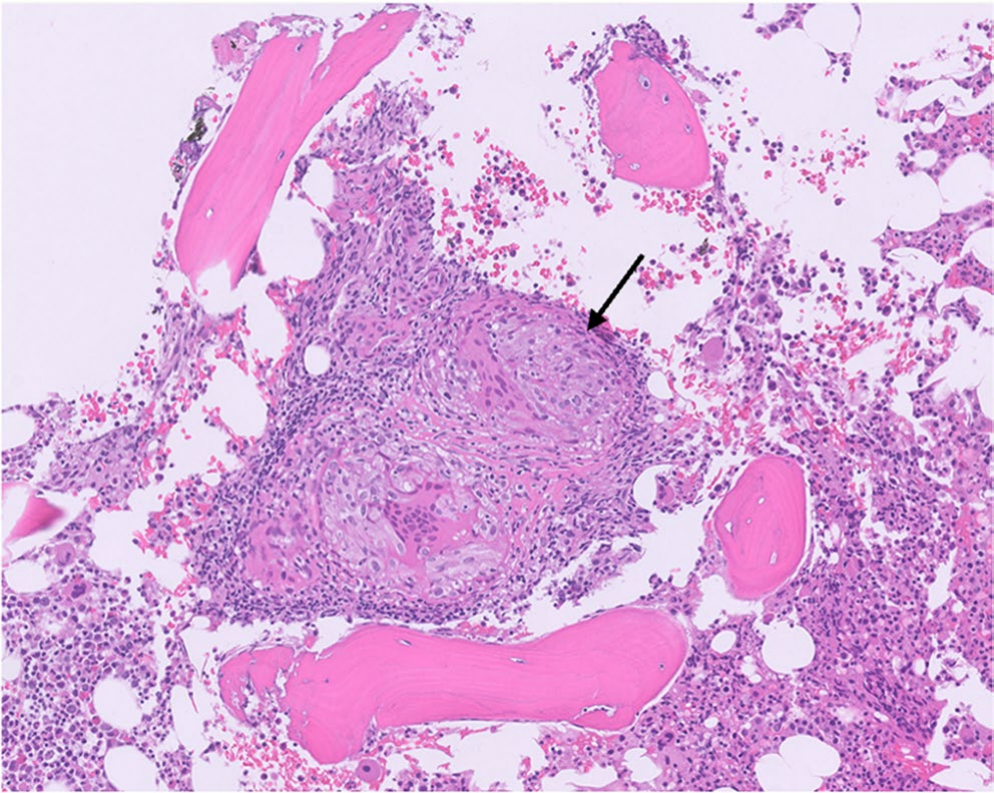
Bone marrow granuloma.

A 70‐year‐old male with a known history of sarcoidosis presented with progressive anemia. The patient was initially diagnosed with sarcoidosis twenty years prior in the setting of parotid gland swelling with biopsy revealing non‐caseating granulomas. At that time, he was found to be pancytopenic; bone marrow biopsy showed hypocellularity consistent with aplastic anemia and increased CD8+ T‐cells and a clonal peak by TCRB PCR indicative of T‐LGLL, but no granulomas. He was treated with anti‐thymocyte globulin (ATG) and long‐term cyclosporine with good response. His baseline labs included WBC 1.4 K/μL, Hgb 7.4 g/dL, Plts 11 K/μL. His ACE and 1,25 vitamin D level were normal. With treatment, his leukopenia normalized, thrombocytopenia improved (117 to 137 K/μL), and his hemoglobin remained stable in the 11.6 to 13.5 g/dL range. Five years later, the patient developed dyspnea and chest CT imaging showed a micronodular pattern with mediastinal adenopathy (Figure 1). His cytopenias were stable at this time. He was therefore started on moderate dose steroid treatment followed by a taper with improvement in both his respiratory symptoms and radiographic findings. His parotid gland and lung symptoms remained stable for many years on five mg of prednisone daily. Over the following decade, attempts to taper steroids below five milligrams daily, as well as his cyclosporine, resulted in recurrent anemia (Hgb range 7.2–9 g/dL) and thrombocytopenia (Plts 11–28 K/μL). A repeat bone marrow biopsy, 10 years after initial diagnosis, revealed persistence of T‐LGLL (with a similar TCRB PCR clone, increased CD7(dim)+CD26−CD16+CD8+T‐cells and abnormal TCR‐Vb spectratyping by flow cytometry with no STAT3 or STAT5 mutation detected), but no evidence of granulomas. Attempts to taper his low dose prednisone again resulted in recurrent anemia and another bone marrow biopsy, now 20 years after his initial diagnosis, identified persistent low‐level T‐LGLL and notably scattered non‐caseating sarcoid granulomas in the bone marrow (Image 1). While anemia is a recognized adverse effect of cyclosporine, his hemoglobin had remained stable on this medication for nearly 20 years, and the onset of anemia occurred only after tapering his prednisone. Reintroduction of five mg of prednisone has resulted in improvement in his anemia. The patient had not received other disease‐modifying therapies during his disease course, such as methotrexate or TNF inhibitors. Although the option of initiating anti‐TNF therapy was discussed as an alternative to prolonged corticosteroid use, he elected to remain on a low dose of prednisone, which he had tolerated well for many years.

## 3 Discussion

Sarcoidosis can involve bone marrow leading to hematologic abnormalities such as anemia, leukopenia, and thrombocytopenia. Hematologic abnormalities can be due to direct granulomatous infiltration of the bone marrow, spleen, lymph nodes, or related to immune dysfunction [2]. The association between sarcoidosis and hematologic manifestations such as aplastic anemia and T‐LGLL has been rarely reported, although T‐LGLL is known to have a high concordance with autoimmune disease, at over 30% [3]. There has been one previously reported case of aplastic anemia as a presenting feature of sarcoidosis [4]. This case described a young female who presented with pancytopenia and interstitial lung disease without mediastinal adenopathy. A bone marrow biopsy revealed aplastic anemia, and the patient responded to ATG and cyclosporine. After bone marrow recovery a transbronchial biopsy was performed which revealed noncaseating granulomas. Additionally, rare case reports describe pure red cell aplasia in patients with sarcoidosis, though these were attributed to infection or medication [5, 6]. The mechanism of involvement related to aplastic anemia in sarcoidosis is unclear, but potential explanations include immune mediated destruction of hematopoietic cells, T cell dysregulation leading to bone marrow suppression and granulomatous involvement leading to pancytopenia [7]. Despite its rarity, aplastic anemia should be considered among the range of hematologic manifestations of sarcoidosis.

The association between sarcoidosis and T‐cell Large Granular Lymphocytic (T‐LGL) leukemia is exceedingly rare. A small study by Karakantza et al. highlighted the association between sarcoidosis and lymphoid malignancies which included the first documented case of T‐LGLL in a patient with sarcoidosis [8]. A population‐based cohort study by Patt et al. found a significant association between sarcoidosis and hematologic malignancies, with an increased odds ratio for lymphoma [9]. The study included 24,000 patients with sarcoidosis and found sarcoidosis patients had a significantly higher prevalence of malignancies compared to controls. The association remained significant for both hematologic and solid organ malignancies. The strongest association was observed with lymphoma, particularly within the first year of sarcoidosis diagnosis. This supports the notion that sarcoidosis patients are at a higher risk for developing lymphoproliferative disorders, likely including T‐LGLL. The pathogenesis linking sarcoidosis to lymphoproliferative disorders, including T‐LGLL, likely involves chronic inflammation and immune dysregulation, which may promote malignant transformation of lymphocytes [10]. This highlights the importance of vigilant monitoring for hematologic malignancies in patients with sarcoidosis. Bone marrow biopsy and flow cytometry are crucial to distinguish between sarcoid‐related marrow infiltration and T‐LGLL.

## 4 Conclusion

This case highlights the complex relationship between sarcoidosis and hematologic complications, emphasizing the potential for granulomatous involvement of the bone marrow and its association with conditions like aplastic anemia and T‐LGLL. Corticosteroids remain the primary treatment option, while immunosuppressive agents may be effective in refractory cases. Additionally, while T‐LGLL has long been associated with autoimmune diseases, with up to 37% of T‐LGLL patients having a concomitant autoimmune disease [3], it is rarely reported to be associated with sarcoidosis. Long‐term monitoring and a multidisciplinary approach are crucial to optimizing outcomes in patients with sarcoidosis and hematologic involvement.

## Author Contributions

**Nina Couette:** conceptualization, writing – original draft, writing – review and editing. **Zachary Braunstein:** conceptualization, writing – original draft, writing – review and editing. **Daniel Jones:** conceptualization, writing – review and editing. **Jonathan E. Brammer:** conceptualization, writing – review and editing. **Arindam Singha:** conceptualization, writing – review and editing.

## Funding

The authors have nothing to report.

## Consent

Written informed consent was obtained from the patient for publication of this case report and any accompanying images.

## Conflicts of Interest

The authors declare no conflicts of interest.

## Data Availability

The authors have nothing to report.
